# Association of arterial blood pressure and CPR quality in a child using three different compression techniques, a case report

**DOI:** 10.1186/1757-7241-21-51

**Published:** 2013-07-02

**Authors:** Marko Sainio, Robert M Sutton, Heini Huhtala, Joar Eilevstjønn, Jyrki Tenhunen, Klaus T Olkkola, Vinay M Nadkarni, Sanna Hoppu

**Affiliations:** 1 Department of Intensive Care Medicine, Critical Care Medicine Research Group, Tampere University Hospital and University of Tampere, PO Box 2000, Tampere, FI-33521, Finland; 2 Department of Anesthesiology, Critical Care and Pediatrics, The Children's Hospital of Philadelphia, University of Pennsylvania School of Medicine, 34th Street and Civic Center Boulevard, Philadelphia, PA, 19104, USA; 3 Lecturer in Biostatistics, School of Health Sciences, University of Tampere, Tampere, FI-33014, Finland; 4 Laerdal Medical AS, PO Box 377, Stavanger, N-4002, Norway; 5 Department of Surgical Sciences, Anaesthesiology and Intensive Care, Uppsala University, Uppsala, SE 751 85, Sweden; 6 Department of Anaesthesiology, Intensive Care, Emergency Care and Pain Medicine, University of Turku and Turku University Hospital, Kiinamyllynkatu 4-8, PO Box 52, Turku, FI-20521, Finland; 7 Department of Emergency Medicine, Department of Intensive Care Medicine and Emergency Medical Services, Critical Care Medicine Research Group, Tampere University Hospital and University of Tampere, PO Box 2000, Tampere, FI-33521, Finland

**Keywords:** Cardiac Arrest, Child, Quality, CPR

## Abstract

A 2-year-old boy found in cardiac arrest secondary to drowning received standard CPR for 35 minutes and was transported to a tertiary hospital for rewarming from hypothermia.

Chest compressions in hospital were started using two-thumb encircling hands technique. Subsequently two-thumbs direct sternal compression technique and after sternal force/depth sensor placement, chest compression with classic one-hand technique were done. By using CPR recording/feedback defibrillator, quantitative CPR quality data and invasive arterial pressures were available for analyses for 5 hours and 35 minutes.

316 compressions with the two-thumb encircling hands technique provided a mean (SD) systolic arterial pressure (SAP) of 24 (4) mmHg, mean arterial pressure (MAP) 18 (3) and diastolic arterial pressure (DAP) of 15 (3) mmHg. ~6000 compressions with the two thumbs direct compression technique created a mean SAP of 45 (7) mmHg, MAP 35 (4) mmHg and DAP of 30 (3) mmHg. ~20,000 compressions with the sternal accelerometer in place produced SAP 50 (10) mmHg, MAP 32 (5) mmHg and DAP 24 (4) mmHg.

Restoration of spontaneous circulation (ROSC) was achieved at the point when the child achieved normothermia by using peritoneal dialysis. Unfortunately, the child died ten hours after ROSC without any signs of neurological recovery.

This case demonstrates improved hemodynamic parameters with classic one-handed technique with real-time quantitative quality of CPR feedback compared to either the two-thumbs encircling hands or two-thumbs direct sternal compression techniques. We speculate that the improved arterial pressures were related to improved chest compression depth when a real-time CPR recording/feedback device was deployed.

**Trial registration:**

ClinicalTrials.gov: NCT00951704.

## Introduction

A previously healthy 21 month-old child was found floating in a small lake at a home playground approximately 5–10 min after submersion. Layperson bystander CPR was initiated and the emergency ambulance reached the child within ~15-20 minutes after submersion. The initial documented rhythm on the pre-hospital resuscitation record was asystole. After initial BLS efforts (i.e., bag mask ventilation and provision of chest compressions (CCs), a tracheal tube was placed approximately 15–20 minutes into the resuscitation. Attempts to obtain intravenous access in the field were unsuccessful. The ambulance staff was instructed to continue resuscitation efforts by the referring emergency physician due to presumed hypothermia, with the plan to rewarm the child by peritoneal dialysis upon arrival to the emergency room (ER). The child arrived to the emergency room approximately 65 minutes after the initiation of out-of-hospital chest compressions.

In the ER, the intensive care unit (ICU) resuscitation team, led by two intensivist physicians, took the responsibility for the resuscitation. The HeartStart MRx with Q-CPR™ option (CPR sensor without the screen on the sensor), jointly designed by Philips Health Care (Andover, MA) and the Laerdal Medical Corporation (Stavanger, Norway) was connected to the child to monitor CPR quality and cardiac rhythm. Ventilations rate target were 10-12/min and was controlled via EtCO2 monitoring by the HeartStart MRx. Venous access was obtained via two intraosseus needles. Approximately 20 minutes after arrival to the ER, an invasive arterial line was also placed. The child’s initial temperature was 26°C when he arrived on the ER. A total of six doses of epinephrine (0.1 mg) were given in the ER before the child’s temperature was known and before the invasive pressure measurement was started. Further doses were not given until normothermia was achieved.

The CCs were first done by using the two-thumb encircling hands technique on the lower half of the sternum. Very soon after the invasive arterial pressure measurement was started, the rescuer changed compression style from the two-thumb encircling hands technique to the two-thumbs direct sternal compression technique due to subjective fatigue. This two thumbs direct sternal compression technique, similar to a classic one-hand compression technique, used the thumbs of both hands to compress the sternum downward (without encircling the childs chest with the hands). Because the invasive arterial pressure attained seemed to improve with this technique, the resuscitation was continued with this method. Given that the HeartStart MRx with Q-CPR™ option was monitoring the child from the initial moments of the resuscitation and it is not easy to have adequate compression depth in child of this age, the team decided to try using the Q-CPR compression sensor (sternal accelerometer / force transducer) to further monitor CPR quality. Deployment of the compression sensor / real time audio-visual feedback device coupled with transition to the classic one-hand technique resulted in improved invasive arterial pressures. The resuscitation attempt was continued with the compression sensor in place until normothermia was achieved and spontaneous circulation was returned. ROSC was achieved 6 hours and 40 minutes after cardiac arrest and lasted ten hours.

## Methods

For this case report, there was no need for ethics approval according to Finnish practice, but written permission to analyze and report the results was obtained from the parents of the child and from the head of the department, and this study is in compliance with the Helsinki Declaration. Quantitative CPR quality data (e.g., rate, depth, incomplete chest wall recoil) and invasive arterial pressures were available for analysis during the 5 hours and 35 minutes that the Q-CPR option was deployed.

Patient episode was analyzed using a Windows-based program, QCPR-Review (v2.1.1.0, Laerdal Medical Corporation, Stavanger, Norway), and Matlab R2010a (Mathworks, Natick, MA). Each compression was detected and analyzed for compression depth and peak systolic and diastolic blood pressure. The diastolic pressure was defined as the lowest inflection point at the beginning of the next compression upstroke. The results were stored and further analyzed using Excel (Microsoft, Redmond, WA) and statistical analysis was performed with SPSS 16.0 (SPSS Inc., Chicago, IL).

The resuscitation attempt was divided into 1-min epochs for evaluation. Outcome variables of interest included overall average compression depth (mm), compression rate (CC/min), chest compression force (kg), and compression count (actual number of compressions delivered per minute), as well as the event no flow fraction (NFF: fraction of time that compressions were not performed during the cardiac arrest). Further, percentages of epochs with average depth < 40 mm or average rates < 90 or > 120 min^-1^ were also determined. Incomplete chest release was evaluated by as a percentage of the total chest compressions delivered and as median incomplete chest release force (kg).

Standard descriptive statistics were utilized and included number and percentage of compressions, mean ± standard deviation (SD), range represented as minimum and maximum or median and 25% to 75% interquartile range (IQR) as determined by the underlying distribution of the data. A oneway analysis of variance was used to determine the relationship between compression depth and invasive arterial pressure. P-values less than 0.05 were considered significant.

## Results

### Quantitative CPR Data

Overall, 25,159 compressions were recorded by the Q-CPR option and available for analysis. Invasive pressure data was available for 316 compressions performed with the 2-thumbs encircling hands technique, 5 569 compressions with the two thumbs direct sternal compression technique, and 19 581 compressions with the sternal accelerometer in place. The Anterior-Posterior (AP) chest depth of this 2 year old (13 kg) child at autopsy was 100 mm. During the time when a sternal accelerometer was used the mean compression depth was 43 (6) mm; 28% of epochs had an average depth < 40 mm (40% AP chest depth). Mean chest compression force was 27 (7) kg. Mean compression rate was 115 (6) CC/min; 2% of epochs had an average rate < 90 or > 120 CC/min. The mean compression count was 113 (7) CC/min. No flow time was only 2% of total resuscitation time when a sternal accelerometer was used. Only 1% of compressions had incomplete chest release, and median incomplete chest release force 0.7 (0.3, 1.3) kg. The child was resuscitated in the ER’s bed with thin, firm mattress (4–5 cm) with a backboard in place Table 1.

**Table 1:** Data on Quality of Cardiopulmonary Resuscitation (CPR)

**Variable**	**Finding**
Episode duration, (h:min:s)	3:40:50
Total number of compressions (n)	25159
Chest compression depth (mm)	43 (6)
Fraction of 1 min epochs with compression depth < 40 mm, No (%)	63/222 (28)
Chest compression force (kg)^a^	27 (7)
Chest compression rate (min^-1^)^b^	115 (6)
Fraction of 1 min epochs with compression rate <90 or >120 min^−1^, No (%)	4/222 (2)
Compressions delivered, (min^-1^)^c^	113 (7)
Incomplete chest release of total number of compressions, No (%)^c^	288/25159 (1)
Incomplete chest release per 1 min epochs, %^d^	0 (0, 0)
Incomplete chest release force, (kg)^e^	0.7 (0.3, 1.3)
Compression as part of duty cycle, %	42 (5)
No flow fraction^f^	0.02 (0.05)
No flow time per 1 min epochs, s^f^	1 (3)

The data are presented as number and percentage, as median and 25 % to 75 % interquartile range (IQR), or as mean and standard deviation (SD) as appropriate (non-normal or normal distribution, respectively).

^a^Indicates the force to needed to the chest compression.

^b^Indicates the rate of compressions when delivered.

^c^Indicates the average number of compressions actually given per minute during the resuscitation attempt.

^d^Indicates the incomplete chest wall decompression after chest compression phase.

^e^Indicates the residual lean weight (leaning force) after chest compression phase.

^f^Indicates the proportion of time without chest compressions during the resuscitation attempt.

### Physiologic Data

**Figure 1:**
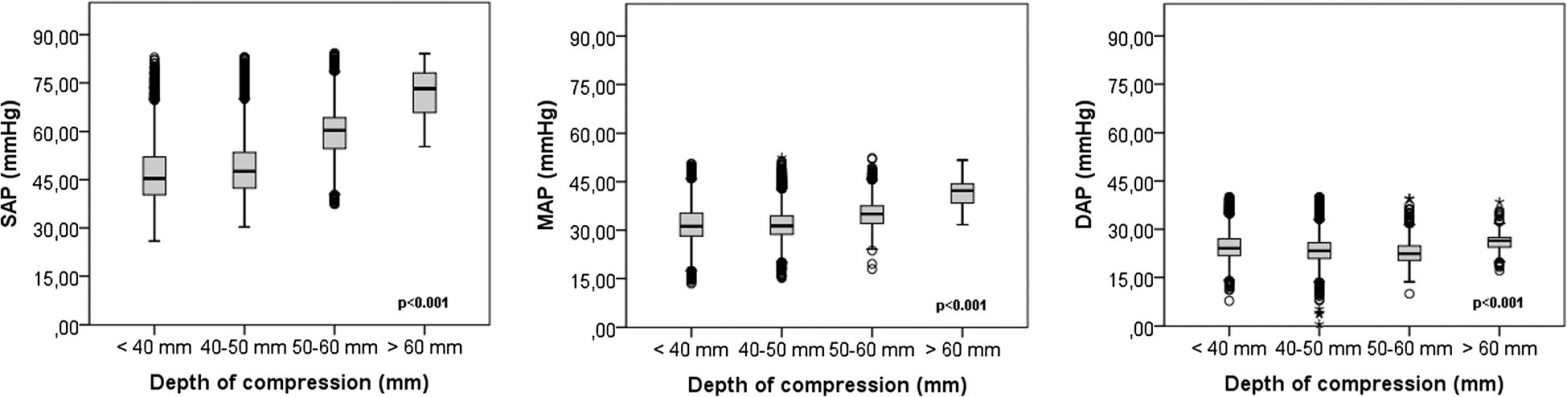
Association of Invasive BP from an arterial catheter with chest compression technique.

The 2-thumbs encircling hands technique imparted a mean systolic arterial pressure (SAP) of 24 (4) mmHg (n = 316), mean arterial pressure (MAP) of 18 (3) mmHg (n = 293) and diastolic arterial pressure (DAP) of 15 (3) mmHg (n = 293). During this time, mean (SD) end-tidal carbon dioxide concentration (EtCO_2_) was 3.0 (1) kPa, (range 1.7-4.4 kPa). The two thumbs direct sternal compression technique resulted in a mean SAP of 45 (7) mmHg (n = 5 569), MAP of 35 (4) mmHg (n = 5 569) and DAP of 30 (3) mmHg (n = 5 861). During this time mean EtCO_2_ was 2.8 (0.5) kPa (range 2.0-4.0 kPa). The one-hand compression technique with a sternal accelerometer applied to the chest with real-time feedback imparted a mean SAP of 50 (10) mmHg (n = 19 581), MAP of 32 (5) mmHg (n = 19 581) and diastolic arterial pressure (DAP) of 24 (4) mmHg (n = 19 353). During this time mean EtCO2 was 2.7 (1) kPa (range 1.0-3.8 kPa) SAP was highest with the sternal accelerometer and real-time feedback compared to other CPR techniques, but DAP was highest with the two thumbs direct sternal compression technique; p < 0.001 (Table 2, Figure 1).

**Table 2:** **Invasive arterial pressures and end-tidal CO**_**2 **_**(EtCO**_**2**_**) during cardiopulmonary resuscitation**

	**SAP (mmHg)**^**a**^	**MAP (mmHg)**^**a**^	**DAP (mmHg)**^**a**^	**EtCO**_**2**_^**a, b**^
2-thumbs encircling hands technique (n ~ 300)	24 (4)	18 (3)	15 (3)	3.0 (1) [1.7-4.4]
Two thumbs direct compression technique (n ~ 5 500)	45 (7)	35 (4)	30 (3)	2.8 (0.5) [1.9-3.6]
Compression with a sternal accelerometer (n ~ 19 500)	50 (10)	32 (5)	24 (4)	2.7 (1) [1.0-3.8]
< 40 mm	47 (9) (n = 6 882)	32 (6) (n = 6 882)	25 (4) (n = 6 770)	Not available
40-50 mm	49 (9) (n = 9 572)	32 (5) (n = 9 572)	24 (4) (n = 9 461)	Not available
50-60 mm	60 (8) (n = 2 860)	35 (4) (n = 2 860)	23 (3) (n = 2 857)	Not available
> 60 mm	72 (7) (n = 253)	41 (4) (n = 253)	26 (3) (n = 253)	Not available

^a^The data are presented as mean and standard deviation (SD) or as mean and (SD) and minimum-maximum [range] as appropriate.

^b^The values are presented as kPa.

### Compression-to-compression analysis with sternal accelerometer

**Figure 2:**
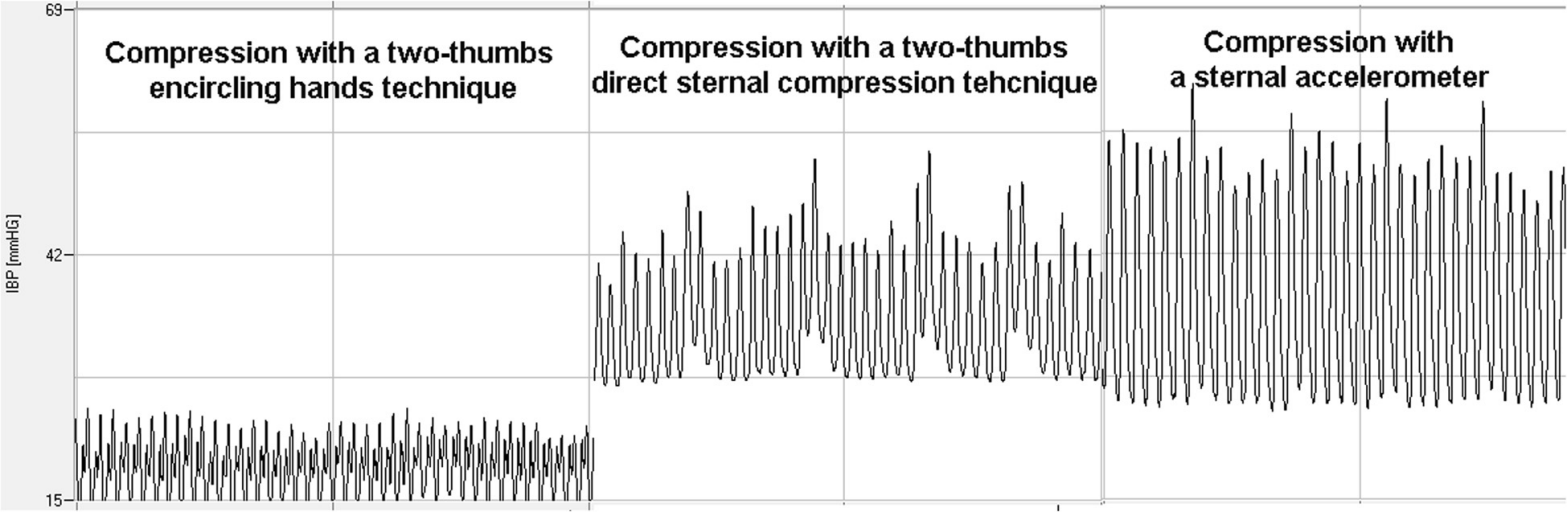
Association of Invasive BP from an arterial catheter with chest compression depth.

Compression depths < 40 mm, 40–50 mm, 50–60 mm and > 60 mm created; SAP of 47 (9), 49 (9), 60 (8), 72 (7) mmHg; MAP of 32 (6), 32 (5), 35 (4), 41 (4) mmHg; and DAP of 25 (4), 24 (4), 23 (3), 26 (3) mmHg respectively (p < 0.001) (Table 2, Figure 2).

The AP chest diameter of this child was 100 mm at autopsy, so compressions should be at least 33 mm (one third) to 50 mm (one half ). In this case the compression depths approximately 1/3 AP chest depth (30–36 mm,n = 1 806) and ½ AP chest depth (47–53 mm, (n = 2 421) created SAP 47 (10) and 53 (8) mmHg respectively (p < 0.001).

### Autopsy findings

The coroner’s autopsy searched for, but did not find any evidence of common possible resuscitation injuries including rib fractures, sterna/xiphoid fracture, cardiac contusions, hemothorax, cardiac tamponade, or liver lacerations.

## Discussion

This case report gives us important information on the relationship between chest compression quality/technique and the attained blood pressure. A 2-thumbs encircling hands technique, usually recommended for infant resuscitation if there are two or more rescuers, did not create acceptable pressures, and was difficult to perform in a child of 2 years age. The direct sternal compression technique with the tips of two fingers, are also recommended in infants for the lone rescuer situation. In this case the two thumbs (two fingers) direct sternal compression technique resulted in rapid achievement of adequate blood pressure when compared it to the 2-thumbs encircling hands technique. Specifically, diastolic arterial pressure and exhaled CO2 were well maintained. When compressions were continued with a one-hand technique supplemented by sternal accelerometer and force transducer real-time guidance, the CPR quality was excellent, and the systolic arterial pressures were even higher. Although speculative, this could be due to the wider spread of pressure over the thoracic cage or, perhaps more likely, to the feedback signal from the defibrillator guiding CPR quality, especially the compression depth and rate and full-release. In the compression-to-compression analysis there was a significant correlation with invasive blood pressures and compression depth.

Current resuscitation guidelines for a small infant recommend a 2-thumb encircling hands or 2 finger sternal chest compression techniques. For child over one year either 1- or 2-hand technique is recommended [1-3]. The sternum should be pressed at least one third of the anterior-posterior (AP) chest diameter in all children, which means approximately 40 mm (1.5 inches) in infants and 50 mm (2 inches) in children [4]. In this case, the increase in compression depth from 1/3 to ½ AP chest diameter created higher SAP.

During adult advanced life support the use of feedback devices during CPR are encouraged, since they improve rescuer ability to achieve correct chest compression depth, rate and chest recoil. The feedback devices also attempt to minimize no-flow time, as much as possible [5,6]. Although the quality deficiencies are similar in adult and pediatric resuscitation attempts, the feedback devices currently available are recommended for children at least 8 years of age. In this case the quality of resuscitation attempt was controlled by invasive pressure. To achieve better pressure team wanted to check CPR quality by using the Q-CPR compression sensor, and since it seemed to result in improved invasive arterial pressures the resuscitation attempt was continued with the compression sensor in place until spontaneous circulation was returned.

The quality of the CPR and compliance with current guidelines was good in this case, at least in those later phases when it was controlled. We do not have similar data describing the quality of CPR before the Q-CPR compression sensor was deployed. Previous studies have shown that the compression depth in children is correlated to invasive pressures [7], but depth itself very seldom reaches the targeted level [8]. This may have happened also in the current case before the use of compression sensor. The mean compression depth in our patient with compression sensor was 43 (6) mm.

There is evidence that the sternal accelerometer that calculated the movement of the Q-CPR sensor, even for a child on a mattress with backboard, may include some movement of the mattress. Therefore our presented depths are higher than the actual compression of the chest achieved. However, the amount of depth movement attributable to a thin, hard stretcher mattress is minimal [9].

In this case deployment of the feedback device improved the invasive blood pressure, and we hypothesize that this was related to improved chest compression quality since no epinephrine was given until normothermia was achieved. Future studies will be needed to further determine the relationship between quantitatively measured depths and blood pressure during actual pediatric resuscitation attempts.

## Conclusions

This case demonstrates that improved hemodynamic measurements were attained using a classic one-handed technique with real-time quantitative quality of CPR feedback compared to either the two-thumbs encircling hands or two-thumbs direct sternal compression techniques. We speculate that the improved arterial pressures were related to improved chest compression depth when a CPR recording / feedback device was deployed in this pediatric cardiac arrest victim.

## Consent

For this case report, there was no need for ethics approval according to Finnish practice, but written inform consent was obtained from the child's parents and from the haed of the hospital for the publication of this report and any accompanying images.

## Abbreviations

SAP: Systolic arterial pressure; MAP: Mean arterial pressure; DAP: Diastolic arterial pressure.

## Competing interests

Dr. Robert Sutton is supported through a career development award from the Eunice Kennedy Shriver National Institute of Child Health & Human Development (K23HD062629). Dr. Jyrki Tenhunen has been a member of international advisory board for SuPARnostic (Virogates, Copenhagen, Denmark) and is CMO and shareholder in SenSem Technologies Ltd (Tampere, Finland.) and Medieta Ltd (Helsinki, Finland). Joar Eilevstjønn is employees of Laerdal Medical AS, Stavanger, Norway. Dr. Vinay Nadkarni is supported from the Laerdal Foundation for Acute Care Medicine. Dr. Sanna Hoppu has provided paid consultancy for Laerdal Medical Corporation. Laerdal Medical has not funded any part of this study, nor had access to any of the data or partaken in the process of the study. Other authors have no conflicts of interest to declare.

## Authors’ contributions

MS had full access to all of the data in the study and takes responsibility for the integrity of the data and the accuracy of the data analysis. Study concept and design: MS, SH, RMS, VMN. Critical revision of the manuscript for important intellectual content: MS, RMS, VMN, JT, KTO, SH. Analysis and interpretation of data: MS, HH, SH, RMS, VMN, JE. Statistical analysis: MS, HH. All authors read and approved the final manuscript.
